# Identification of key genes with abnormal RNA methylation modification and selected m6A regulators in ankylosing spondylitis

**DOI:** 10.1002/iid3.1314

**Published:** 2024-08-02

**Authors:** Fengqing Wu, Hongbin Huang, Deyang Sun, Bingbing Cai, Huateng Zhou, Renfu Quan, Huan Yang

**Affiliations:** ^1^ Department of Orthopedics Yiwu Central Hospital Yiwu China; ^2^ Department of Orthopedics Second Affiliated Hospital of Chongqing Medical University Chongqing China; ^3^ First College of Clinical Medicine Zhejiang Chinese Medical University Hangzhou China; ^4^ Department of Orthopedics Hangzhou Xiaoshan District Chinese Medicine Hospital Hangzhou China; ^5^ Department of Biochemistry Zhejiang University School of Medicine and Zhejiang University Medical Center Hangzhou China

**Keywords:** ankylosing spondylitis, bioinformatics, N6‐methyladenosine (m6A), RNA sequence analyses

## Abstract

**Background:**

N6‐methyladenosine (m6A) has been identified as the most abundant modification of RNA molecules and the aberrant m6A modifications have been associated with the development of autoimmune diseases. However, the role of m6A modification in ankylosing spondylitis (AS) has not been adequately investigated. Therefore, we aimed to explore the significance of m6A regulator‐mediated RNA methylation in AS.

**Methods:**

The methylated RNA immunoprecipitation sequencing (meRIP‐seq) and digital RNA sequencing (Digital RNA‐seq) were conducted using the peripheral blood mononuclear cells from three AS cases and three healthy controls, to identify genes affected by abnormal RNA methylation. The genes associated with different peaks were cross‐referenced with AS‐related genes obtained from the GeneCards Suite. Subsequently, the expression levels of shared differentially expressed genes (DEGs) and key m6A regulators in AS were evaluated using data from 68 AS cases and 36 healthy controls from two data sets (GSE25101 and GSE73754). In addition, the results were validated through quantitative polymerase chain reaction (qPCR).

**Results:**

The meRIP‐seq and Digital RNA‐seq analyses identified 28 genes with upregulated m6A peaks but with downregulated expression, and 52 genes with downregulated m6A peaks but with upregulated expression. By intersecting the genes associated with different peaks with 2184 AS‐related genes from the GeneCards Suite, we identified a total of five shared DEGs: *BCL11B*, *KAT6B*, *IL1R1*, *TRIB1*, and *ALDH2*. Through analysis of the data sets and qPCR, we found that *BCL11B* and *IL1R1* were differentially expressed in AS. Moreover, two key m6A regulators, *WTAP* and heterogeneous nuclear ribonucleoprotein C, were identified.

**Conclusions:**

In conclusion, the current study revealed that m6A modification plays a crucial role in AS and might hence provide a new treatment strategy for AS disease.

## Graphical abstract

This article reveals that aberrant expression of m6A regulators contributes to AS development. This relation may be conducted by genes *BCL11B* and *IL1R1*, as well as m6A regulators *WTAP* and *HNRNPC*. Considering the importance of m6A modification as a critical regulator on AS, blocking or inhibiting certain m6A enzymes may reveal previously unidentified strategies for therapeutic interventions against AS disease.

## 1 INTRODUCTION

Ankylosing spondylitis (AS) is a common, chronic, and autoimmune disease that involves axial joints and entheses.^1^ Globally, the prevalence of AS is estimated to range between 0.1% and 1.4% and has a gender ratio of 2:1 (male: female).^2^ However, currently, there is no effective cure for AS, although immunosuppressants especially interleukin (IL)‐17 inhibitors have shown good potential to alleviate disease progression,^3^ but with a corresponding increase in the risk of nonserious infections, which range from mild to moderate, and hence increasing medical costs of the disease management.^4^ Therefore, it is imperative to investigate the pathogenesis and establish better treatment methods of AS. Both environmental factors and genetic susceptibility contribute to the development of AS disease.^5^ Furthermore, through genome wide association studies, it has been shown that 90% of all AS cases are associated with *HLA‐B27*,^6^ and *ERAP1* mutations only affect the susceptibility of the patients with *HLA‐B27* alleles.^7^, ^8^ Through high‐throughput sequencing methods, multiple susceptibility genes have been found to participate in the genetic pathogenesis of AS.^9^

Recently, an increasing number of studies have been conducted with a focus on the field of epigenetics to explain how genes interact with the environment and thus affect the process of AS disease.^10^, ^11^, ^12^ Epigenetics mainly includes DNA methylation, histone marks, and RNA modification whereby the methylation of the N (6) position of adenosine (m6A) is the most common RNA modification on eukaryotic messenger RNA (mRNA). In addition, the RNA methylation is involved in translation, degradation, splicing, and export of mRNA metabolism, that extensively affect mammalian development, cell differentiation, immunity, metabolism, and tumors among other life processes. Besides, m6A modification has also been identified for its important biological functions in regulating noncoding RNAs such as microRNAs, circular RNAs, and long noncoding RNAs (lncRNAs).^13^ The N6‐methyladenosine (m6A) methylated‐related enzymes are divided into methyltransferases (writers), demethylases (erasers), and methylated reading proteins (readers).^14^ The main function of writers is to “enter” m6A methylation into a specific RNA site to achieve RNA methylation modification. In contrast, the role of “erasers” is to demethylate the RNA that have been modified with m6A. On the other hand, “readers” are required for m6A‐modified RNA to perform specific biological functions through specifically recognizing m6A modified mRNA, promoting RNA translation, or maintaining the stability of RNA.

Accumulating evidence show that m6A modification participates in a variety of disease processes and particularly plays vital role in autoimmune diseases.^15^ Furthermore, several studies of m6A have been applied on various autoimmune diseases and immune related cells with the goal of discovering new therapies.^16^, ^17^ Currently, several studies have explored the significance of m6A regulators.^18^, ^19^, ^20^ Moreover, it has been found that *METTL14*‐dependent m6A modification of *ELMO1* contributes to the directed migration of mesenchymal stem cells in AS.^21^ Furthermore, *METTL14* was found to be downregulated in T cells from patients with AS, resulting in impaired autophagic flux and severe inflammation due to reduced *FOXO3a* expression.^22^ However, the relationship between AS and m6A is not well understood. Therefore, this study preliminarily attempted to evaluate the potential role and mechanism of m6A methylation regulators in AS to generate ideas for guiding clinical diagnosis and treatment of the disease.

## 2 MATERIALS AND METHODS

### 2.1 Sample collection and RNA extraction

Samples were selected from The Affiliated Jiangnan Hospital of Zhejiang Chinese Medical University using a random sampling method. The current study was reviewed by the Ethics Committee of Hangzhou Xiaoshan District Chinese Medicine Hospital and also met all the requirements of the Declaration of Helsinki of the World Medical Assembly. All participants provided signed informed consent forms to participate in the study. Peripheral blood mononuclear cells (PBMCs) were acquired from whole blood of the participants through density gradient centrifugation and total RNA of PBMCs were extracted with Invitrogen's TRizol^TM^ Reagent.

Three AS cases and three controls were then referred to Wuhan Kangce Technology Co., Ltd, for Nano methylated RNA immunoprecipitation sequencing (MeRIP‐seq) and digital mRNA sequencing (Digital mRNA‐seq), while nine AS cases and nine controls were used for quantitative polymerase chain reaction (qPCR) validation.

### 2.2 Nano meRIP‐seq and m6A methylation peak determination

In this study, 5 μg total RNAs were used for meRIP experiment. Total RNAs was extracted using TRIzol Reagent (Invitrogen, cat. no. 15596026). Briefly, 20 mM ZnCl_2_ was added to total RNA and incubated at 94°C for 5 min until the RNA fragments were mainly distributed in 100 nt. Fragmented RNA was precipitated with anti‐m6A antibody (Synaptic Systems, 202203) for m6A immunoprecipitation (m6A‐IP). The stranded RNA sequencing library was constructed by using KC‐DigitalTM Stranded mRNA Library Prep Kit for Illumina® (Catalog no. DR08502, Wuhan Seqhealth Co., Ltd). Then, ribosomal complementary DNA (cDNA) was removed by SMARTer Stranded Total RNA‐Seq Kit version 2 (Pico Input Mammalian; 634413; Takara/Clontech). The quality control of the completed libraries was performed on Novaseq. 6000 sequencer (Illumina) with PE150 model.

Raw sequencing data were filtered by Trimmomatic (version 0.36) and then clustered to eliminate any errors and biases introduced by PCR amplification or sequencing. Package “exomePeak” (Version 3.8) software was used for peak calling and differential analysis. The m6A‐binded regions (also called peaks in each m6A‐IP sample) were later detected using the corresponding m6A‐input sample being the control.^23^ Significant peaks with *p* < .05 and |logFC| > 1 were identified and annotated to the RefSeq database (hg19) using STAR software (version 2.5.3a) with default parameters. Furthermore, HOMER (version 4.9) was utilized to perform peak annotation and to analyze the binding motif. The m6A methylation peak was displayed by integrative genomics viewer (IGV) software according to the BW file of sequencing samples.

### 2.3 Digital mRNA‐seq and differential expressed m6A regulators verification

Two micrograms of total RNAs were used for digital mRNA‐seq library preparation using Ribo‐off rRNA Depletion Kit (Human/Mouse/Rat) (Catalog no. MRZG12324, Illumina) and KC‐DigitalTM Stranded mRNA Library Prep Kit for Illumina® (Catalog no. DR08502, Wuhan Seqhealth Co., Ltd) following the manufacturer's instruction. The library products corresponding to 200–500 bp were enriched, quantified, and finally sequenced on NovaSeq. 6000 sequencer (Illumina) with PE150 model. In the present study, *t* test was used to examine the differential expressed genes with a cutoff of *p* < .05 between the AS group and the normal control group.

Heatmaps were then constructed for 27 m6A regulators and box plots for the potential m6A regulators in AS.

The present study annotated the differential peak with gene symbol and compared it with the DEGs of mRNA‐seq to identify the shared DEGs using the “ggscatter” package.

### 2.4 Data acquisition and preprocessing

The identified gene set was overlapped with the ankylosing‐sondylitis‐related gene list extracted from GeneCards^24^ with the “VennDiagram” package.

Two gene expression matrices were obtained from the Gene Expression Omnibus (GEO) data sets (https://www.ncbi.nlm.nih.gov/geo/). The GSE25101 data set comprises 16 AS cases and 16 healthy controls, whereas GSE73754 consists of 52 AS samples and 20 healthy donors (controls). Both matrices contain samples from peripheral blood. Probe names were converted to gene names using the “affy” package and the “removeBatchEffect” function of the “limma” package was employed to eliminate batch effects in the RStudio environment.

### 2.5 Differential expression analysis of m6A regulators

A total of 27 widely recognized m6A regulators were collected from previously published literatures,^25^, ^26^ including 10 writers (*METTL3, METTL4, METTL14, CBLL1, WTAP, KIAA1429, RBM15B, RBM15, ZC3H13*, and *ZNF217*), two erasers (*ALKBH5* and *FTO*), and 15 readers (*YTHDF1, YTHDF2, YTHDF3, IGF2BP1, IGF2BP2, IGF2BP3, YTHDC1, YTHDC2, EIF3A, EIF3B, HNRNPA2B1*, heterogeneous nuclear ribonucleoprotein C (*HNRNPC*), *LRPPRC, FMR1*, and *ELAVL1*).

Differential expression analysis for these regulators between two groups was performed using wilcox.test and visualized using “ggplot2” package. The level of significance difference was set at *p* < .05. The correlation between the m6A regulators was identified using “corrplot” package. To identify potential m6A RNA methylation regulators in patients with AS disease, univariate logistic regression was conducted and interrupted at *p* < .05. Dimension reduction and variable selection was performed using the least absolute shrinkage and selection operator (LASSO). Further, the risk scores of selected AS‐related genes were also calculated, whereby the box plot and receiver operating characteristic (ROC) curve analysis were adopted, to analyze the prediction effect of the risk model.

### 2.6 qPCR

The quality and purity of the isolated total RNA were tested by Nanodrop one (Thermo Scientific). RNA samples with A260/280 values between 1.8 and 2.1 were used for subsequent analysis. Using Thermo Scientific Revert Aid First Strand cDNA Synthesis Kit (Thermo Scientific), a total of 1 μg of RNA was reverse‐transcribed into cDNA. All primers were supplied by Beijing Liuhe Huada gene technology company. The primers are as follows:

*GAPDH*_F 5′‐GGAGCGAGATCCCTCCAAAAT‐3′,

*GAPDH*_R 5′‐GGCTGTTGTCATACTTCTCATGG‐3′;

*KAT6B*_F 5′‐GCCTTGCCTCCTATAAGGACC‐3′, *KAT6B*_R 5′‐TCCACATTGCGGAGATCATTAC‐3′;

*TRIB1*_F 5′‐GCTGCAAGGTGTTTCCCATTA‐3′, *TRIB1*_R 5′‐TCCCCAAAGTCCTTCTCAAAGA‐3′;

*BCL11B*_F 5′‐TCCAGCTACATTTGCACAACA‐3′, *BCL11B*_R 5′‐GCTCCAGGTAGATGCGGAAG‐3′;

*IL1R1*_F 5′‐ATGAAATTGATGTTCGTCCCTGT‐3′, *IL1R1*_R 5′‐ACCACGCAATAGTAATGTCCTG‐3′;

*WTAP*_F 5′‐CTTCCCAAGAAGGTTCGATTGA‐3′, *WTAP*_R 5′‐TCAGACTCTCTTAGGCCAGTTAC‐3′;

*HNRNPC*_F 5′‐GGAGATGTACGGGTCAGTAACA‐3′, *KAT6B*_R 5′‐CCCGAGCAATAGGAGGAGGA‐3′.

R *GAPDH* was used as an endogenous constant control. Use △△*C*_t_ method to determine the relative difference of mRNA expression between AS patients and healthy controls.^27^

## 3 RESULTS

### 3.1 Conjoint analyses of MeRIP‐seq and RNA‐seq

**Figure 1:**
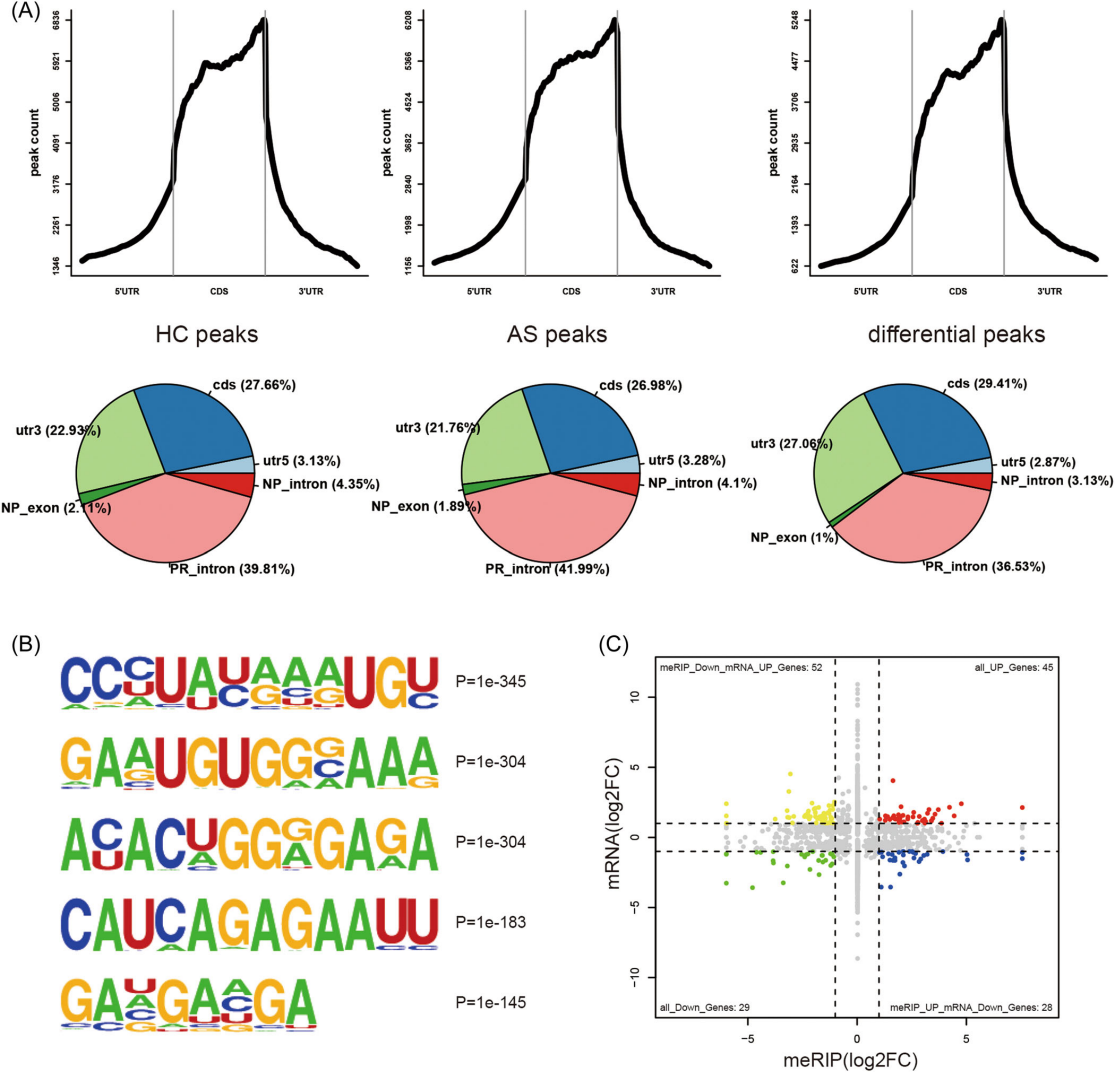
Conjoint analyses of methylated RNA immunoprecipitation sequencing (MeRIP‐seq) and RNA sequencing (RNA‐seq). (A) Distribution of peaks in 5′‐untranslated region (UTR), CDS, 3′‐UTR area (the upper), and distribution statistics of peaks in each functional area of gene (the lower). The proportion of differential peaks distributed in 3′‐UTR and CDS region was a little higher than AS peaks and healthy control (HC) peaks (cds is the CDS region of the gene, utr5 and utr3 are the 5′‐UTR and 3′‐UTR of the gene, respectively. NP_exon is the exon region of noncoding genes, NP_intron is the intron region of noncoding genes, and PR_intron is the intron region) (B). The top five motifs related to differential peaks. (C) Volcano map demonstrated 28 “hyper‐down” genes and 52 “hypo‐up” genes. CDS, coding sequence.

To determine the difference in RNA methylation modification by m6A between AS and healthy controls, the meRIP‐seq method was employed to sequence the methylated RNA of PBMCs from three AS cases and three healthy donors. Results showed that the methylation peaks of AS were significantly different from those in the control group. Moreover, although there was no difference in the overall distribution of methylation peaks between AS cases and the control samples, it was evident that the proportion of differential peaks distributed in 3′‐untranslated region and coding sequence regions was significantly increased (Figure 1A). Furthermore, the current study identified the top five motifs related to differential peaks (Figure 1B). The AS samples had 1337 dysregulated m6A peaks, with 619 m6A peaks significantly upregulated and 718 m6A peaks significantly downregulated. Moreover, we identified 2952 DEGs from UID mRNA‐seq, with 1546 genes significantly upregulated and 1406 genes significantly downregulated. Through conjoint analyses of MeRIP‐seq and RNA‐seq data, we got 28 genes with upregulated m6A peaks but with downregulated expression, referred to as “hyper‐down” genes, and 52 genes with downregulated m6A peaks but with upregulated expression, referred to as “hypo‐up” genes (Figure 1C).

### 3.2 *BCL11B* and *IL1R1* were identified as key genes of AS

**Figure 2:**
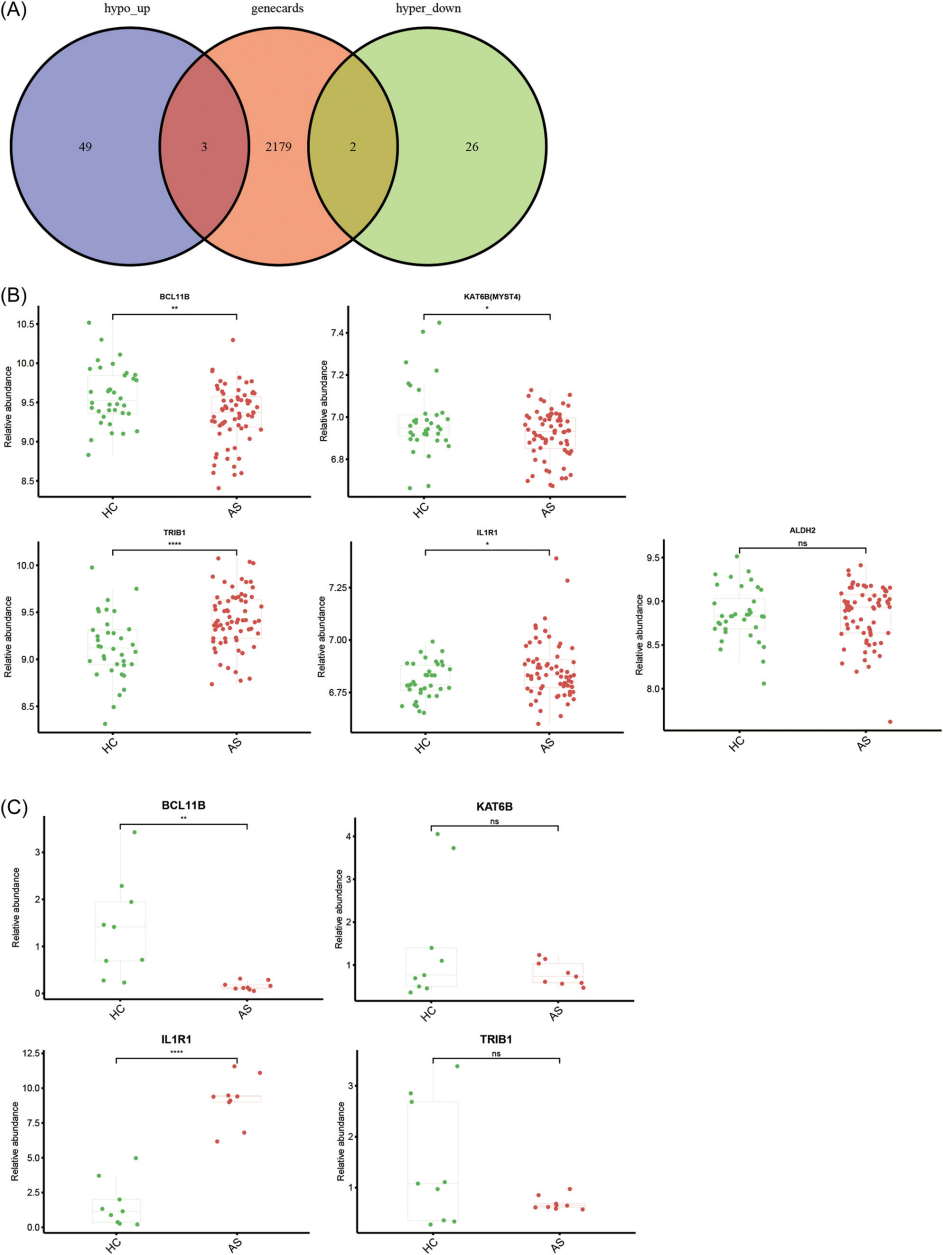
*BCL11B* and *IL1R1* were identified as key genes of ankylosing spondylitis (AS). (A) Venn diagram showed five deifferentially expressed genes (DEGs) shared by different peak related genes and 2184 DEGs from messenger RNA sequencing. (B) The expression of five shared DEGs in Gene Expression Omnibus data sets. (C) The relative expression of four shared DEGs in quantitative polymerase chain reaction.

**Figure 3:**
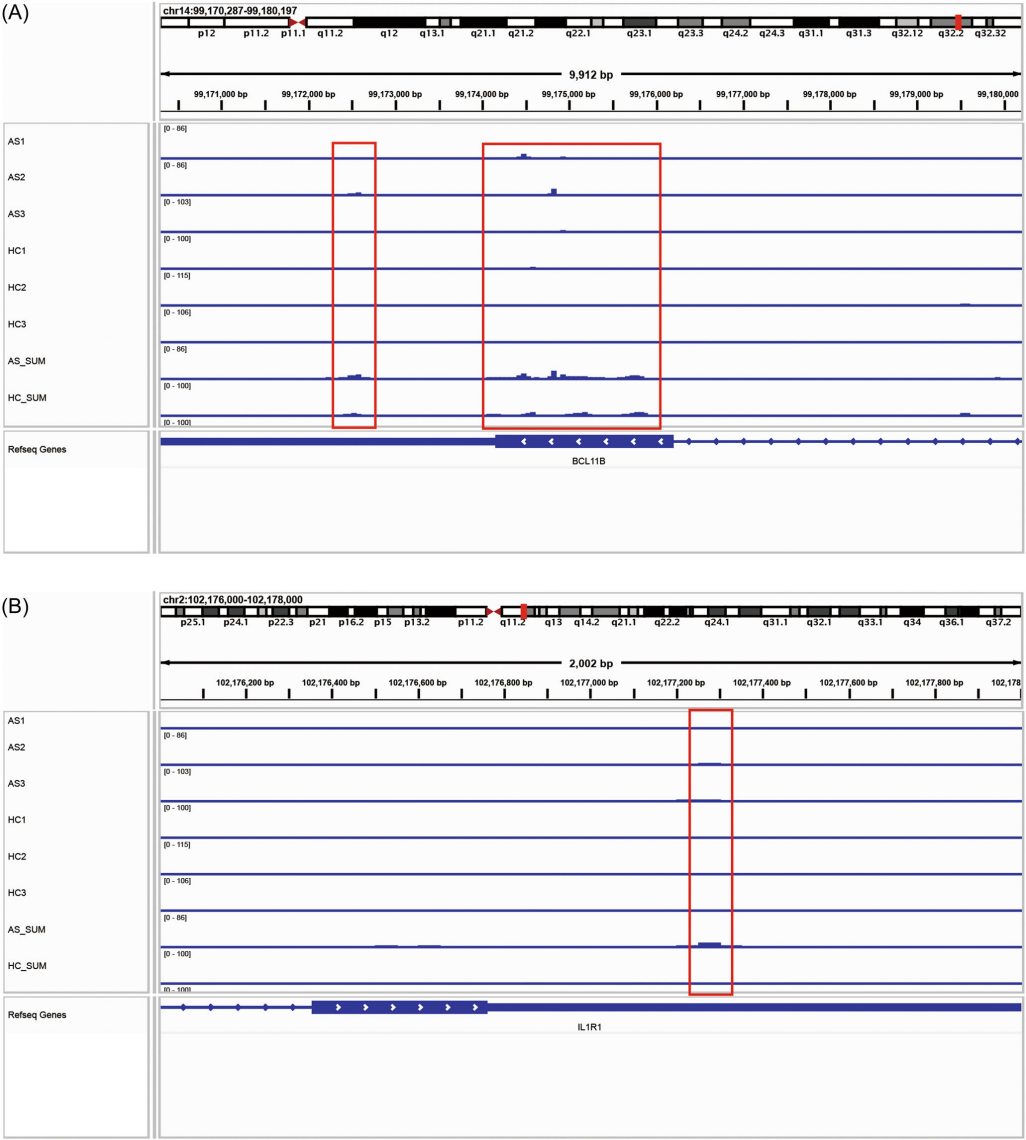
m6A methylation peaks of *BCL11B* and *IL1R1*. (A) Integrative genomics viewer (IGV) plots showing m6A methylated peaks for *BCL11B*. (B) IGV plots showing m6A methylated peaks for *IL1R1*. Blue boxes represent exons and blue lines represent introns.

After intersecting the differentially associated peak genes with 2184 genes related to AS from the GeneCards Suite, a set of five shared DEGs was identified (Figure 2A), namely *BCL11B*, *KAT6B*, *IL1R1*, *TRIB1*, and *ALDH2*. To corroborate these results, the expression levels of these five shared DEGs were assessed in 68 AS cases and 36 healthy controls across two data sets (GSE25101 and GSE73754) using qPCR. The results showed that *BCL11B* and IL1‐receptor 1 (*IL1R1*) were differentially expressed in AS (Figure 2B,C). IGV plots showing different m6A methylated peaks for *BCL11B* and *IL1R1* between AS and healthy control cases (Figure 3A,B).

### 3.3 Abnormal expression of selected m6A regulators in AS

**Figure 4:**
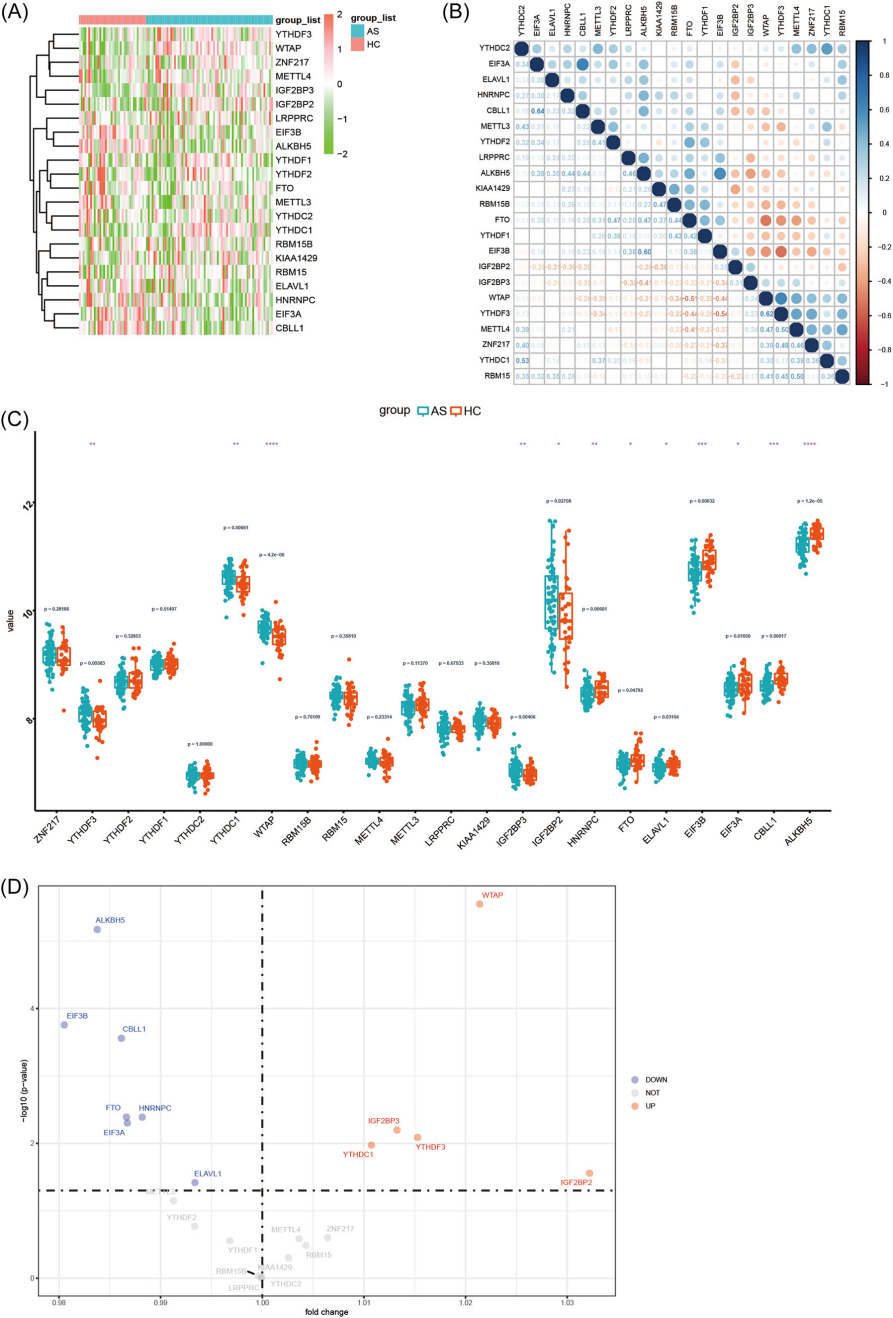
Abnormal expression of selected m6A regulators in ankylosing spondylitis (AS). (A) The Heatmap of the expression differences of 22 m6A RNA methylation regulators between AS cases and healthy controls. (B) Correlations among the expression of 22 m6A regulators in all samples. (C, D) The box plot and Volcano map demonstrated the expression differences of 22 m6A RNA methylation regulators between AS cases and healthy controls. Compared with the healthy controls, the expression of *WTAP*, *YTHDC1*, *YTHDF3*, *IGFBP2*, and *IGFBP3* were upregulated and *HNRNPC*, *EIF3A*, *EIF3B*, *CBLL1*, *ELAVL1*, *FTO*, and *ALKBH5* were downregulated in AS (*p < .05; **p < .01; ***p < .001; ****p < .0001).

By analyzing the differential expression of 27 m6A RNA methylation regulators (22 m6A regulators were actually found in the expression data) between the two groups, a total of 12 m6A RNA methylation regulators were found to be significantly related to AS disease (Figure 4C). The upregulated genes included *YTHDF3*, *YTHDC1*, *WTAP*, *IGF2BP2*, and *IGF2BP3*, whereas the downregulated genes included *EIF3A*, *EIF3B*, *HNRNPC*, *CBLL1*, *ELAVL1*, *ALKBH5*, and *FTO* (Figure 4D).

### 3.4 *WTAP* and *HNRNPC* identified as potential m6A regulators of AS

**Figure 5:**
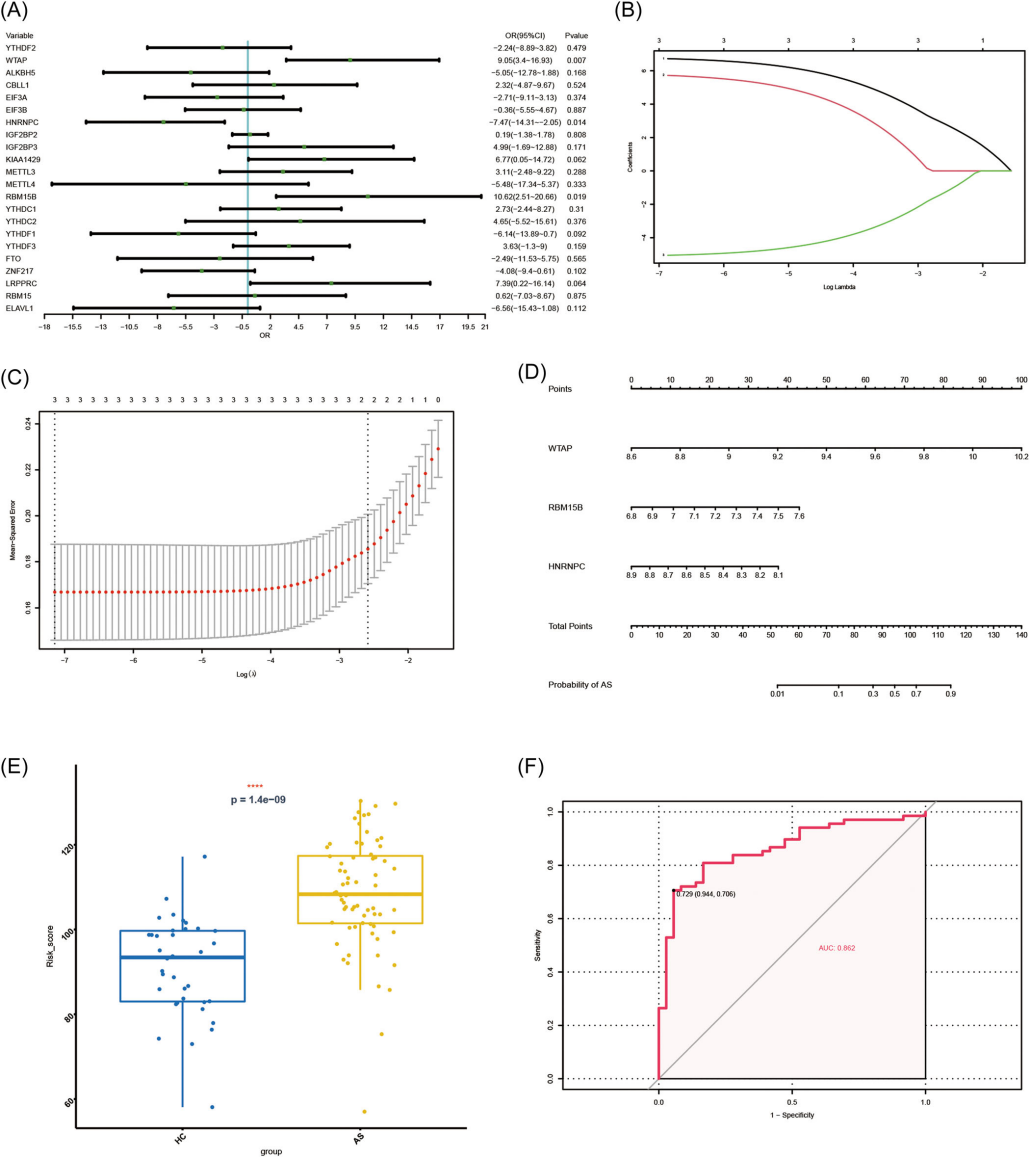
Identification of crucial m6A regulators in ankylosing spondylitis (AS). (A) Univariate logistic regression investigated the relationship between 22 m6A regulators and AS cases, revealing three AS‐related m6A regulators: *WTAP, RBM15B*, and heterogeneous nuclear ribonucleoprotein C (*HNRNPC*) (*p* < .05). (B) Least absolute shrinkage and selection operator (LASSO) coefficient profiles of *WTAP, RBM15B*, and *HNRNPC*. (C) 10‐fold cross‐validation for tuning parameter selection in the LASSO regression. (D) The risk scores of *WTAP* and *HNRNPC*. (E) The risk distribution between healthy and AS cases showed that AS cases have a much higher risk score than healthy controls. (F) The discrimination ability of m6A regulators in healthy and AS cases analyzed by receiver operating characteristic (ROC) curve and evaluated by area under the curve value.

In this study, univariate logistic regression was performed to analyze the expression data of 22 m6A regulators. The findings indicated that *WTAP, RBM15B*, and *HNRNPC* were potential m6A regulators associated with AS disease (Figure 5A). Further, results of LASSO Cox regression indicated that the three m6A regulators were also significant in AS disease (Figure 5B,C). In addition, it was found that the risk scores of the three regulators significantly increased in the AS group with the *p* value being 1.4e−09 (Figure 5D,E). Moreover, the plotted ROC curve revealed that the m6A regulators had significant potential in distinguishing between the AS and control cases (Figure 5F). Ultimately, the *WTAP* and *HNRNPC* were identified as the potential m6A regulators of AS, as their expression had been found to be significantly related in AS.

### 3.5 Verification of *WTAP* and *HNRNPC* as potential m6A regulators of AS

**Figure 6:**
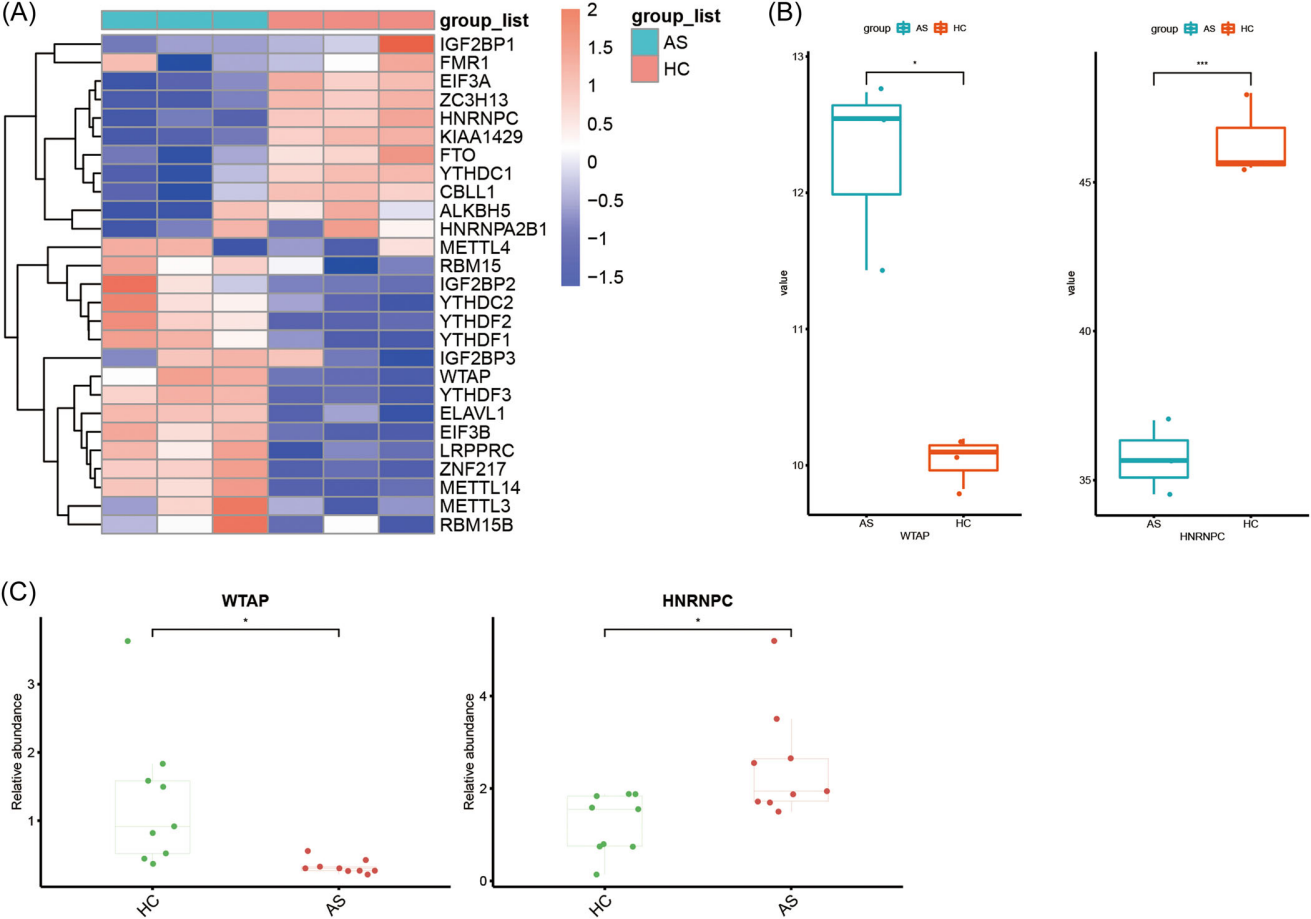
Differentially expressed m6A regulators between three ankylosing spondylitis (AS) cases and three healthy controls. (A) Heatmap for 27 differentially expressed m6A regulators (*p* < .05). (B) Box plots for the expression of *WTAP* and heterogeneous nuclear ribonucleoprotein C (*HNRNPC*) in Gene Expression Omnibus data sets. *WTAP* was significantly upregulated in AS with *HNRNPC* being significantly downregulated. (C) Box plots for the relative expression of *WTAP* and *HNRNPC* in quantitative polymerase chain reaction. *WTAP* was significantly upregulated in AS with *HNRNPC* being significantly downregulated. (*p < .05; **p < .01; ***p < .001; ****p < .0001).

After getting the differential expressed genes between the three AS cases and three healthy control samples, heatmaps (for 27 m6A regulators) and box plots (for *WTAP* and *HNRNPC*) were constructed (Figure 6A). It was evident that *WTAP* was significantly upregulated in AS, whereas *HNRNPC* was significantly downregulated, and the findings were in agreement with results of previous studies (Figure 6B) and qPCR results (Figure 6C).

## 4 DISCUSSION

Recently, research studies have reported that abnormal m6A modifications contribute to the occurrence of autoimmune diseases.^28^, ^29^ Research has demonstrated that m6A‐related enzymes regulate responses to nonmicrobial double‐stranded DNA in uninfected cells, thereby influencing host immunity and potentially contributing to autoimmune diseases.^30^ Further, it is evident that the m6A reader, *IMP2* can direct autoimmune inflammation through an IL‐17‐ and tumor necrosis factor‐α (*TNF‐α*)‐dependent C/EBP transcription factor axis.^31^ Moreover, aberrant m6A modifications has been linked to the initiation of several autoimmune diseases, including rheumatoid arthritis,^32^ systemic lupus erythematosus (SLE),^33^, ^34^ multiple sclerosis,^35^ and AS.

In this study, through conjoint analyses of meRIP‐seq and RNA‐seq, we got 28 “hyper‐down” genes and 52 “hypo‐up” genes, among which *BCL11B* and *IL1R1* were identified as key genes of AS. In addition, two potential m6a regulators, *WTAP* and *HNRNPC*, were identified through bioinformatics analysis and confirmed using qPCR. This indicate that m6A modification contributes to the the occurrence of AS, probably by targeting *BCL11B* and *IL1R1*.

*BCL11B* is a C2H2 zinc finger domain‐containing transcription factor,^36^ which is first expressed at the early stage of thymocytes and modulates the the survival of double‐negative and double‐positive thymocytes, β selection, and positive selection of CD4 and CD8 single‐positive thymocytes.^37^ It has been shown to block the TH2 effector program in T‐helper 1 (TH1) and TH17 cells.^38^ *BCL11B* is essential for maintaining the identity and function of innate lymphoid cells type 2,^39^ and for controlling the antigen‐specific clonal expansion and cytolytic function of CD8^+^ T lymphocytes. Mice lacking *BCL11B* in double‐positive thymocytes develop inflammatory bowel disease, with massive pro‐inflammatory cytokine‐producing CD4^+^ T cells infiltrating in the colon.^37^ This phenomenon was similarly observed in mice deficient in *BCL11B* expression within Treg cells, where a decline in suppressor function was observed, accompanied by decreased levels of *Foxp3* and *IL‐10*, and increased production of pro‐inflammatory cytokines TNF, interferon‐γ, and *IL‐17*.^40^ *BCL11B*/Treg knockout mice presented fatal systemic autoimmunity and died at an early age.^41^ In this study, we identified decreased mRNA levels of the *BCL11B* gene in AS patients, which is consist with previous research.^42^

*IL1R1* is located in the *IL‐1* gene cluster. It can affect nuclear factor‐κB signaling and lead to the upregulation of inflammatory by combining with *IL‐1* on the cell surface.^43^ Cytokines have been implicated in the pathogenesis of AS,^44^ including *IL‐1*, which has been reported to be associated with AS.^45^ *IL1R1* polymorphisms was also found to be an increased risk factor for AS in a Northwest Chinese Han population.^46^ Our research revealed increased mRNA levels of *IL1R1* gene in AS patients, further confirming its relationship with AS.

*WTAP* and *HNRNPC*, essential regulators of m6A methylation, can regulate m6A modifications. Analysis of DEGs in the GSE73754 data set revealed that *WTAP* was upregulated in AS.^20^ The *WTAP* is necessary for nuclear speckle localization and formation of *METTL3* and *METTL14* complex formation, which functions as an m6A methyltransferase and is essential for alternative splicing and gene expression.^47^ The *HNRNPC* protein, which is part of the large family of ubiquitously expressed heterogeneous nuclear ribonucleoproteins, binds to nascent RNA transcripts and affects pre‐mRNA stability, splicing, export, and translation.^48^ M6A modification can alter the local structure of mRNA or lncRNA by facilitating the interaction with specific reader proteins, such as *HNRNPC*. This interaction, in turn, regulates mRNA splicing patterns.^49^ The mRNA levels of *WTAP* has been found to be decreased in peripheral blood from SLE patients, as well as primary Sjogren's syndrome patients.^50^ Our study found that *WTAP* was upregulated in peripheral blood from AS patients, whereas *HNRNPC* was downregulated, indicating abnormal m6A modification in AS may be due to the two regulators. Unfortunately, we did not detect other significant m6A regulators, such as *METTL14*, which have been reported in previous studies.^20^, ^22^ Nonetheless, our study presents the first attempt to integrate multiple analyses, including MeRIP‐seq, Digital RNA‐seq, GeneCards Suite, GEO data sets, and qPCR, thereby identifying crucial genes associated with AS. Therefore, our study provides innovative insights meant to improve the clinical diagnosis and treatment of AS. However, our study had several limitations. First, the sample size used for sequencing and qPCR tests was limited. In addition, we did not confirm the overall methylation level of AS nor the functional role of m6A in AS pathogenesis. Therefore, further investigation and validation are necessary to elucidate the precise association and exact mechanism of m6A modification in AS.

In conclusion, this work reveals that aberrant expression of m6A regulators contributes to AS development. This relation may be conducted by genes *BCL11B* and *IL1R1*, as well as m6A regulators *WTAP* and *HNRNPC*. Therefore, the precise association and exact mechanism of m6A modification in AS disease still needs further investigation and verification. Furthermore, considering the importance of m6A modification as a critical regulator on AS, blocking or inhibiting certain m6A enzymes may reveal previously unidentified strategies for therapeutic interventions against AS disease.

## AUTHOR CONTRIBUTIONS

**Fengqing Wu**: Conceptualization; methodology; data curation; formal analysis; validation; writing—original draft; writing—review and editing; visualization. **Hongbin Huang**: Methodology; resources; data curation; supervision; writing—review and editing. **Deyang Sun**: Software; formal analysis; visualization. **Bingbing Cai, Huateng Zhou**: Resources; data curation. **Renfu Quan**: Conceptualization; project administration; funding acquisition; supervision; resources. **Huan Yang**: Validation; writing—review and editing. All authors revised and approved the manuscript.

## CONFLICT OF INTEREST STATEMENT

The authors declare no conflict of interest.

## ETHICS STATEMENT

Ethics committee approval was received for this study from the Ethics Committee of Hangzhou Xiaoshan District Chinese Medicine Hospital, review batch number: 202012KL‐012. This study conforms to Declaration of Helsinki standards and has obtained the prior informed consent to participate in research as well as publish from all participants.

## Data Availability

The raw data used and analyzed during the current study are available from the first author or the corresponding author on reasonable request. The microarray data generated in this study have been uploaded in the SRA data sets database, with the BioProject ID being PRJNA833263 and PRJNA833405, and will be released on May 31, 2024.
